# An Iterative Leave-One-Out Approach to Outlier Detection in RNA-Seq Data

**DOI:** 10.1371/journal.pone.0125224

**Published:** 2015-06-03

**Authors:** Nysia I. George, John F. Bowyer, Nathaniel M. Crabtree, Ching-Wei Chang

**Affiliations:** 1 Division of Bioinformatics and Biostatistics, National Center for Toxicological Research, FDA, Jefferson, Arkansas, United States of America; 2 Division of Neurotoxicology, National Center for Toxicological Research, FDA, Jefferson, Arkansas, United States of America; 3 Joint Bioinformatics Graduate Program, University of Arkansas at Little Rock and University of Arkansas for Medical Sciences, Little Rock, Arkansas, United States of America; CNRS UMR7622 & University Paris 6 Pierre-et-Marie-Curie, FRANCE

## Abstract

The discrete data structure and large sequencing depth of RNA sequencing (RNA-seq) experiments can often generate outlier read counts in one or more RNA samples within a homogeneous group. Thus, how to identify and manage outlier observations in RNA-seq data is an emerging topic of interest. One of the main objectives in these research efforts is to develop statistical methodology that effectively balances the impact of outlier observations and achieves maximal power for statistical testing. To reach that goal, strengthening the accuracy of outlier detection is an important precursor. Current outlier detection algorithms for RNA-seq data are executed within a testing framework and may be sensitive to sparse data and heavy-tailed distributions. Therefore, we propose a univariate algorithm that utilizes a probabilistic approach to measure the deviation between an observation and the distribution generating the remaining data and implement it within in an iterative leave-one-out design strategy. Analyses of real and simulated RNA-seq data show that the proposed methodology has higher outlier detection rates for both non-normalized and normalized negative binomial distributed data.

## Introduction

The rise of RNA sequencing (RNA-seq) as a competing tool for differential expression analysis has launched considerable efforts to develop methods that effectively model and analyze count data produced by RNA-seq experiments. Unlike microarray experiments, which produce continuous probe intensities, RNA-seq measures RNA content through digital expression profiling by counting the number of sequencing reads that map to a particular feature (e.g. exon, gene, or transcript). Given the dynamic range of RNA-seq data and practically no ceiling for quantification, extreme high counts (i.e. outliers) for a given feature are often present in one or more RNA samples within an experimental group. The presence of outliers substantially limits the power of differential testing [1,2].

RNA-seq counts are influenced by a number of decisions that must be made to generate expression data from total RNA. As a result, outlier read counts may arise from one of many stages, including biological harvesting of RNA, design implementation, and data processing techniques. For example, in animal studies, in order to obtain sufficient levels of RNA to be sequenced, multiple needle punctures might be necessary to acquire enough of the tissue to be sampled from a relatively small body size. In this case, the possibility of collecting a sample from non-target tissues increases, which could potentially affect read counts in a subset of features. On the data preprocessing side, the selected mapping pipeline and library construction also affect read counts. Since outliers may have biological or technical origins, accurately detecting outliers may help a researcher pinpoint their source and ensure data quality.

Normalization is often the initial step to correct for artifacts in measured expression data (see [3] for an overview of different normalization methods). Typically, a scaling normalization method is implemented when the downstream analysis requires count-based statistical analysis. The primary goal of scaling factor normalization is to minimize between-sample variability for invariant genes by adjusting the sequencing depth of each replicate sample. *edgeR* [4] computes a scaling factor for each sample using the trimmed mean of M-values (log ratio of counts in a sample to counts in a reference sample) [5]. Alternatively, *DESeq2* [6] uses the median of the ratio of counts for a sample to the geometric mean of counts over all samples [7]. Despite the advantages of normalization, normalization procedures cannot adjust for all sources of unknown variation as is evidenced by the fact that both *edgeR* and *DESeq2* incorporate outlier detection methods to improve the robustness of differential analysis.

To date, only a handful of existing R packages identify count-based outliers in RNA-seq data analysis. Zhou et al. introduced a robust method of down-weighting extreme values that could be used within existing testing frameworks. In their work, an observation with a large Pearson residual from a fitted negative binomial generalized linear model is attributed a smaller Huber weight [8]. The resulting method, denoted herein as *edgeR-robust*, can be implemented in *edgeR*. *DESeq2* employs Cook’s [9] distance to measure the degree of influence of a single observation on fitted coefficients of a linear model. Although Huber’s estimate presents a robust approach to down-weight deviant expressions, its sensitivity to extreme outliers can potentially hinder accurate outlier detection in skewed data. Cook’s distance uses a regression-based method to identify influential observations, and thus also may not be optimal for sparse or skewed data.

*edgeR-robust* and Cook’s distance are both carried out within their respective testing methodologies. Thus, we consider isolating read count outliers outside of a test-based strategy. In this work, outlier detection is implemented via a univariate algorithm that is built on two concepts: probabilities associated with the assumed null distribution and a leave-one-out (LOO) iterative scheme. Information from the sequencing depth is used as a criterion to identify observations that deviate substantially from other observations in the same treatment group. Current count-based analysis methods obligate raw (non-normalized) counts and, apart from turning off outlier detection completely, provide no means of separating outlier detection and DE testing. For widespread application, we outline a general algorithm for detecting outliers in raw counts prior to testing. Slight modifications are made to the proposed algorithm in order to effectively compare our results with those obtained from *edgeR* and *DESeq2* methods. We demonstrate the advantages of an iterative LOO (*iLOO*) outlier detection methodology and explore its strengths in detecting multiple feature-level outliers with the use of real and simulated RNA-seq data.

## Methods

### iLOO: iterative leave-one-out approach to outlier detection

Let *Y*_*g*,*i*_ denote the *i* ^*th*^ read count of feature *g* for a given treatment group comprised of *n* samples.

The *iLOO* algorithm is outlined as follows:

1. Estimate the sequencing depth. The point estimate for sequencing depth, $\hat{d},$ is the average total number of reads across each sample, i.e. $\hat{d}=\frac{1}{n}\sum_{i=1}^{n}\sum_{g=1}^{G}Y_{g,i}$, where *G* is the number of features and *n* is the number of samples.
  For each feature *g*,
2. Construct a matrix $X_{n\times \left(n-1\right)}^{g}$, where the rows of the matrix are denoted by $X_{k^{*}}^{g}$. For *k* = 1,…,*n*, build *X* ^*g*^ such that $X_{k^{*}}^{g}=Y_{g,-k},$ where *Y*_*g*,−*k*_ is the vector *Y*_*g*,*i*_ with the *k* ^*th*^ observation removed.
  For each *k* in $X_{k^{*}}^{g}$,
3. Estimate the sample mean $\bar{x}$ and the sample variance *s* ^2^ of *Y*_*g*,−*k*_ using method of moments.
  - Based on the estimated mean-variance relationship, consider one of two distribution families for $X_{k^{*}}^{g}$. If $s^{2}>\bar{x},$ fit a *NB*(*μ*, *ϕ*) distribution to the data and estimate $\hat{\mu }$ and $\hat{\phi }$. Otherwise, fit a *Poisson*(*λ*) distribution to the data and estimate $\hat{\lambda }$.
  - Compute *p*(*y*_*g*,*k*_) based on the model fitted in *a)*.
4. Classify all *y*_*g*,*k*_ observations such that $p\left(y_{g,k}\right)<1/\hat{d}$ as outliers.
5. Remove all *y*_*g*,*k*_ identified in step *4* from *Y*_*g*,*k*_.
6. Repeat steps *2*. through *5*. until all $p\left(y_{g,k}\right)\geq 1/\hat{d}$.

**Fig 1:**
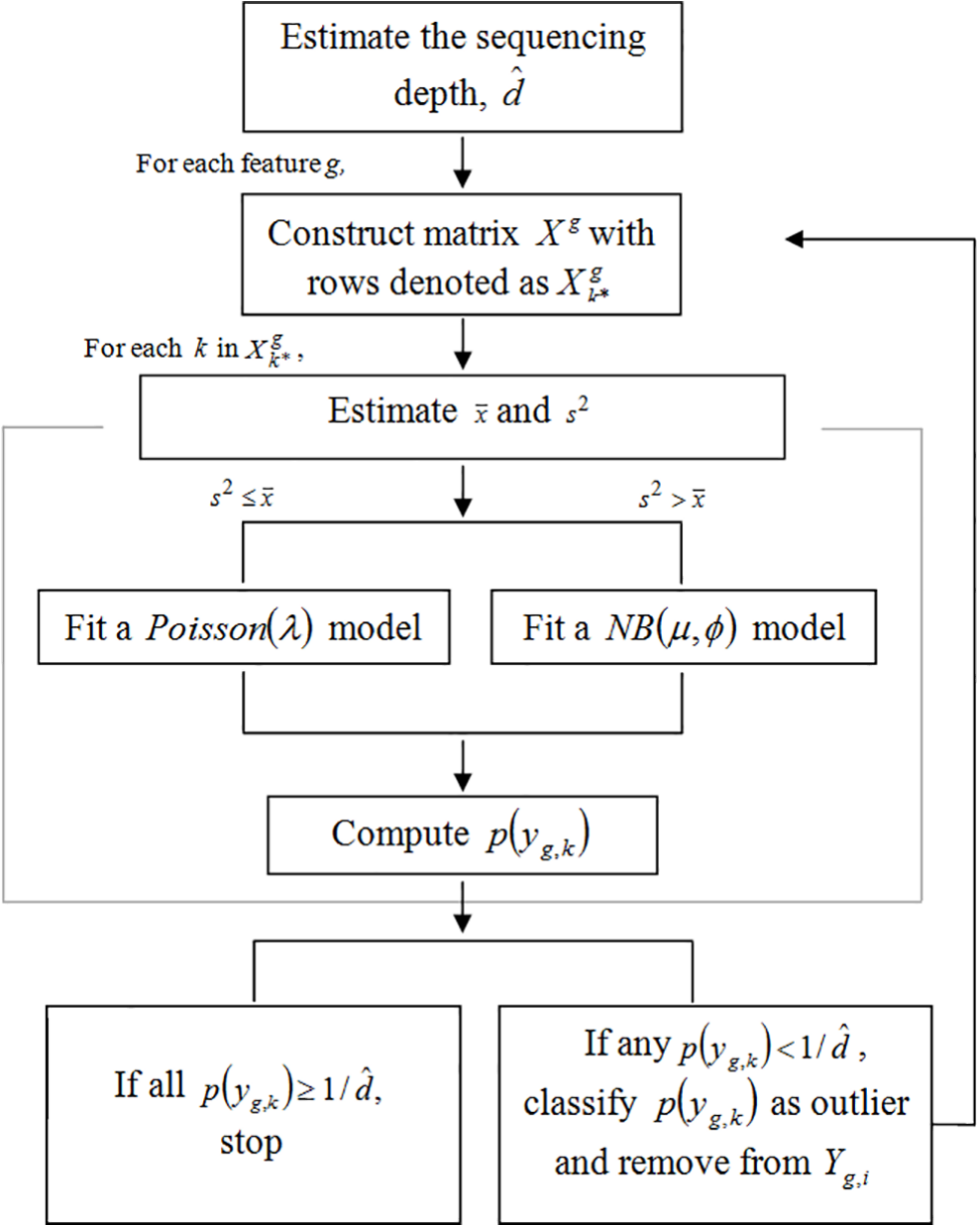
Flowchart of the iLOO algorithm.

A flowchart of the algorithm is provided in Fig 1.

Sequencing depth provides an intuitive basis for the interpretation of whether RNA-seq counts should be classified as outliers. Theoretically, the inverse of the sequencing depth, $1/\hat{d},$ is the minimum empirical observed probability for a sequenced read. Thus, it is used as a criterion to flag deviant expression values.

All analyses were performed using R version 3.1.0 (www.r-project.org). R code for running iLOO is provided as S1 R Code.

### Datasets

Two independent datasets were used to compare outlier methods on real data. The first dataset (GSE62368) consists of RNA sequencing data from whole blood in 85 to 90-day-old male Sprague-Dawley rats obtained from the breeding colony of the National Center for Toxicological Research (NCTR). The study was carried out in accordance with the declaration of Helsinki and the Guide for the Care and Use of Laboratory Animals as adopted and promulgated by the National Institutes of Health. The use of animal testing in this study was done under protocols E7295 and E7519 (issued to John Bowyer) that were approved by the NCTR Institutional Animal Care and Use Committee (IACUC), which is fully accredited (Food and Drug Administration—National Center for Toxicological Research, Accreditation #A4310-01) by NIH-OLAW. Rats were sacrificed at 4 to 5pm with an overdose of 150 mg/kg body weight of pentobarbital, which resulted in deep anesthesia within 3 min. After anesthesia, 3 to 5 ml of cardiac blood was withdrawn with an 18 gauge needle attached to a 5 ml syringe. Rats were euthanized with decapitation. Approximately 1 ml of the blood sample (to be used for RNA-seq analysis) was separated into 300 to 200 aliquots, frozen on dry ice in cylindrical foil capsules, and stored at -70°C. RNA samples were sent to Expression Analysis Inc. [EA; Durham, NC]. EA created the library and performed 50bp paired-end and strand-specific sequencing using an Illumina platform. A total of 25–35 million reads were generated per sample. Raw reads were aligned to the rat reference genome. The full experiment included multiple treatments; however, we focused only on the control group (*n* = 16). Since the experimental treatments produced a great deal of variability in feature-level reads, the control group was used because the data was more stably expressed and should contain fewer outliers. This provides the best platform for evaluating outlier detection without additional effects. A total of 16,895 features were available for analysis after removing features (transcripts, in this study) containing only 0 counts.

The second dataset (*n* = 6) was selected to compare the performance of both methodologies in smaller sample size (and a different RNA-seq pipeline). RNA-seq data from six unrelated cerebellar cortex tissue samples sequenced by Wang et al. [10] was downloaded from ReCount [11]. A total of 11,081 non-zero features were analyzed.

### Simulation study

RNA-seq datasets were simulated to mimic real data using the R package *npSeq* [1]. Under the default setting for simulating negative binomial distributed data, mean expressions are randomly drawn from a source dataset [12] and the dispersion parameter is fixed at 0.25. To relax the constraints imposed by constant dispersion, we modified the dispersion parameter and randomly sampled dispersion from *Gamma*(0.21,1.73) [13]. The concept of independently modeling dispersion with a gamma distribution was previously supported by Hardcastle and Kelly [14].

RNA-seq data for a single class was obtained by selecting the “twoclass” option in *npSeq* and setting the sample size of the second class equal to zero. The default values for number of features, percentage of significant features, and percentage of up-regulated significant features remained unchanged at 20000, 0.3, and 0.8, respectively. Outliers were added to the simulated dataset in a manner similar to the random method outlined by Soneson and Delorenzi [15]. Specifically, 10% of the features (i.e. 2000 features) were randomly selected as outlier features. For each outlier feature, an observation was chosen at random and replaced with *c* × max{*Y*_*g*,*i*_}, where *c* is a random sample from the uniform distribution *U*(5,20). This process was repeated 100 times. Data were generated for sample sizes of *n* = 5,10,15,20,30, and 40 to represent a single treatment group.

## Results

Outliers in real data were assessed with *iLOO* and *edgeR-robust* (version 3.6.8). One of the aims of outlier detection is to identify potential problems with data quality; hence, outlier detection rates for real data were evaluated at the raw data level. Since *DESeq2* performs analysis on normalized counts, it was omitted from real data analysis. An advantage of *iLOO* is its ability to return multiple outlier reads per feature. To make *iLOO* and *edgeR-robust* comparable, we adjusted the single outlier restriction implemented by Zhou et al. [2] and set the outlier cutoff for *edgeR-robust* to be the 10^th^ percentile of down-weighted Huber weights (*r* < 0.50). This adjustment allows the algorithm flexibility to return multiple extreme down-weighted observations per feature. In the *edgeR-robust* methodology, multiple observations with high counts for a given feature can and will be re-weighted to dampen adverse effects on expression analysis so the suggested modification is reasonable. In addition, $\hat{d}$ in Step 1 of the proposed *iLOO* algorithm was modified to reflect the mean of normalized sequencing depths (i.e. effective library sizes in *edgeR*).

**Fig 2:**
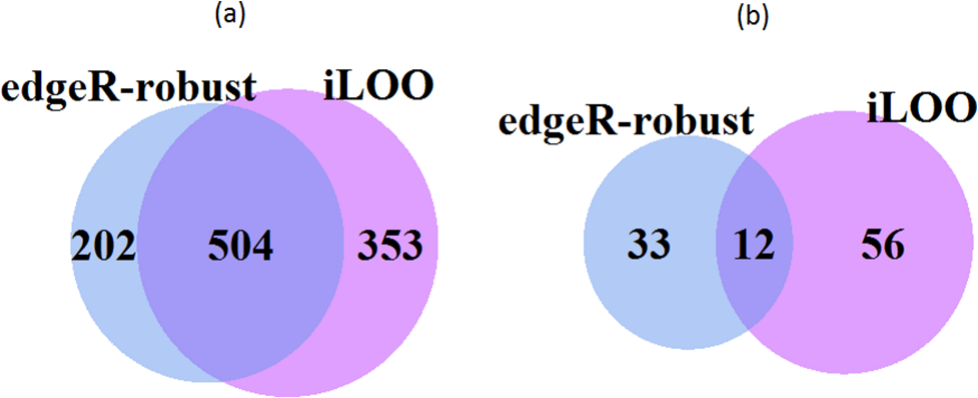
Venn diagram of the number of features with outliers detected by *iLOO* and *edgeR-robust*. The totals provided present the number of (a) single outlier features and (b) features with two detected outliers identified by *iLOO* and *edgeR-robust* in the control group of rat RNA-seq data.

Several summary statistics are reported for our analysis of the rat RNA-seq control group data. The total number of detected outliers by both methods is provided in Table 1. Both methods detected no outliers in almost 95% of the features. Outlier rates for the control animals ranged between 0.5–4%. All samples were below the maximum outlier rate of 10% reported by Zhou et al. In general, *iLOO* detected more outliers than *edgeR-robust* and also more outliers per feature. There was some overlap between *iLOO* and *edgeR-robust* in their identification of features with a single outlier count and features with two detected outliers. However, both methods uniquely classify read counts as outliers (Fig 2).

**Table 1:** Number of features with 0 through 4 detected outliers in the control group of rat RNA-seq data.

No. of Outliers	*iLOO*	*edgeR-robust*
**0**	15967	16144
**1**	857	706
**2**	68	45
**3**	2	0
**4**	1	0

**Fig 3:**
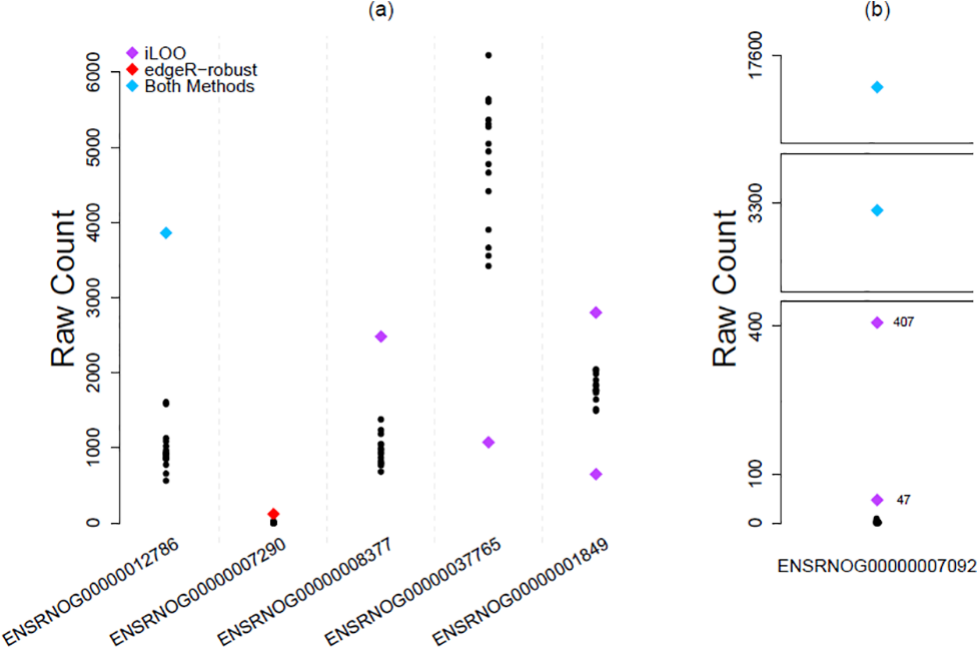
Scatterplot of read counts observed in real data for a sample of features. Scatterplot of raw counts for six representative features displaying counts identified as outliers by *iLOO* (purple diamond), *edgeR-robust* (red diamond), and both methods (blue diamond) in the control group of rat RNA-seq data.

To draw conclusions about the effectiveness of both methods to detect aberrant observations, a scatterplot of raw RNA-seq counts for 6 features with outlier read counts is presented in Fig 3. Noticeably, both methods classify outliers similarly when the non-outlier data has minimal variability and the aberrant observation is considerably distant. When the non-outlier observations show no variability (e.g. feature ENSRNOG00000007290), *edgeR-robust* uniquely identifies an outlier count. In general, the scatter of expression data presented in Fig 3 highlights a number of key findings about the advantages of *iLOO* as an outlier detection methodology. When expression data is more variable (e.g. feature ENSRNOG00000037765), *iLOO* has more power to detect outlying expressions. In addition, *iLOO*’s ability to detect down-regulated outlier counts is an important observation since down-regulated expressions are typically masked in most outlier methodologies. Lastly, Fig 3(B) presents the scatter of expression data for a feature where *iLOO* detected four outlier read counts (feature ENSRNOG00000007092). Both methodologies were able to detect the two counts with extremely high values. However, *iLOO* detected two additional read counts. Given the range of expression for this feature in the bottom split panel of Fig 3(B), count 407 is likely an outlier and count 47 may also be a legitimate outlier considering the remainder of the data is very tightly controlled with a minimum count of 0 and a maximum count of 9.

To show that these properties hold for smaller datasets (and varying RNA-seq pipelines), we applied the same analysis to the cerebellar cortex tissue samples from six unrelated donors [10]. Both methods classify observations identically for 99.7% of the features (S1 Table). However, each method is unique in how it classifies observations as outliers (S1 Fig). The pattern of outlier detection for features that were classified differently parallel our findings from the rat RNA-seq control data. When the non-outlier observations are tightly controlled, both methods identify outliers with ease. Again, we find that *iLOO* is more robust at identifying distant observations and multiple observations per feature, especially when the feature-level data is variable (S2 Fig). The latter is a key point when attempting to identify outliers in a skewed distribution.

### Simulated data analysis

To preserve the integrity of comparisons across methods, separate simulations were carried out to compare *iLOO* with *edgeR* and *iLOO* with *DESeq2* (version 1.4.5). Although *edgeR* and *DESeq2* use similar normalization techniques, they differ in that scaling factors for *edgeR* apply to the sequencing depths and those for *DESeq2* to read counts. Thus, in the first simulation study, we compare *edgeR-robust* to *iLOO (*with modified $\hat{d}$ to account for effective library sizes). *DESeq2* normalizes read counts and makes them available to the user; therefore, *iLOO* was performed on normalized read counts and compared to *DESeq2*. No further adjustment to $\hat{d}$ was necessary since $\hat{d}$ was computed from normalized data.

The metrics used to evaluate the performance of *iLOO*, *edgeR-robust*, and *DESeq2* on simulated RNA-seq data were total accuracy, outlier accuracy, and non-outlier accuracy. Here, accuracy is defined at the feature level. In other words, for a given feature, every observation must be correctly identified as either an outlier or non-outlier in order for that feature to be scored as a success. Total accuracy is defined as the proportion of success features (out of a total of 20,000 features). Outlier accuracy is defined as the number of success outlier features divided by 2000. Likewise, non-outlier accuracy is defined as the proportion of success non-outlier features. Consideration of all three metrics provides the most thorough assessment.

The mean and standard deviation of accuracies computed from the *edgeR-robust* simulation study are presented in Table 2. The findings show that even with a sample size of 5, *iLOO* achieves almost 80% accuracy for all three measures of accuracy. For sample sizes of 10 and higher, total, outlier, and non-outlier accuracy exceed 97%. Although total accuracy for *edgeR-robust* exceeds 90% at all sample sizes, the metric is predominately inflated by non-outlier accuracy. Results of the simulation study suggest that both methods perform reasonably well in minimizing the number of false positive calls; however, *edgeR-robust* appears to struggle to detect true outliers in small and moderate sample sizes. Results of the simulation study comparing *iLOO* (with normalized counts) to *DESeq2* show similar findings (S2 Table).

**Table 2:** Mean (standard deviation) of accuracy metrics from simulated RNA-seq data comparing *iLOO* (using *edgeR* normalized sequencing depths) to *edgeR-robust*.

Method	Accuracy	Sample Size					
		5	10	15	20	30	40
***iLOO***	*Total*	0.9260 (0.0525)	0.9756 (0.0372)	0.9797 (0.0297)	0.9867 (0.0195)	0.9899 (0.019)	0.9885 (0.0176)
	*Outlier*	0.7801 (0.0203)	0.9703 (0.044)	0.9774 (0.0325)	0.9862 (0.0203)	0.9898 (0.0199)	0.9887 (0.0173)
	*Non-Outlier*	0.9422 (0.0575)	0.9762 (0.0364)	0.9799 (0.0294)	0.9867 (0.0194)	0.9899 (0.0189)	0.9884 (0.0177)
***edgeR-robust***	*Total*	0.9118 (0.0039)	0.9244 (0.0118)	0.9371 (0.0195)	0.9486 (0.0238)	0.9512 (0.0213)	0.9515 (0.0283)
	*Outlier*	0.1256 (0.0287)	0.2440 (0.1194)	0.3743 (0.2001)	0.4954 (0.2521)	0.5770 (0.2662)	0.6753 (0.2332)
	*Non-Outlier*	0.9991 (0.0023)	0.9999 (0.0003)	0.9997 (0.0017)	0.9989 (0.0044)	0.9927 (0.0185)	0.9822 (0.0376)

## Discussion

Improving the accuracy of outlier detection is an important step within an RNA-seq data analysis pipeline. It is well known that undetected outliers are certain to mask true statistical significance. It has even been suggested that some methodologies have a tendency to favor outlier features [1]. Furthermore, even if outliers are re-weighted and pulled toward the dispersion-mean trend or replaced with the trimmed mean, the estimated variability for a feature will be affected if detection is not accurate. For example, a high false positive detection rate will result in a biased and underestimated variance, which will ultimately influence differential testing.

The challenge of identifying outliers in discrete data is compounded when the sample size is small. Previous research has shown that moment based estimators are more stable than maximum likelihood estimates for small sample size [16]. Thus, one means to mitigate the effect of small sample size is to use method of moments to estimate parameters of the fitted distribution, which we followed in the current study. The minimum number of replicates in our simulation study was 5. Although many real studies are designed and implemented with sample sizes smaller than 5, outlier detection in such studies may not be meaningful or accurate. *DESeq2* also treats outlier detection in small-sample problems with caution—when the sample size for a group is between 3 and 6, genes with outliers are simply flagged and no p-value is computed. *DESeq2* does not perform outlier detection when the number of replicates is smaller than 3.

Some simulation studies for RNA-seq data use real data as a source dataset and randomly draw joint observations of mean and dispersion [2,17]. While this approach is appropriate when evaluating statistical methodology for expression analysis, it would likely present biases in a study where the researcher has to maintain control of the level of perturbation. It was essential that we simulate the underlying dataset void of the influence of outliers, which was successfully carried out with the use of *npSeq*. Despite the limitations, conclusions drawn from the simulation study closely mirror those obtained from the analysis of real RNA-seq data.

*iLOO* demonstrated superior performance over existing methods in both simulation studies. The gain in terms of increased outlier accuracy was magnitudes higher at smaller sample sizes. Like many previous studies, normalization methods affected study outcomes. In this study, applying *iLOO* to normalized read counts maintained 90% accuracy even at sample size of 5. However, we do not emphasize this approach since it is impossible to integrate outlier-corrected normalized counts into current frameworks that explicitly require raw counts. Nevertheless, our findings highlight the robustness of *iLOO* on normalized and non-normalized negative binomial distributed data.

Outliers in RNA-seq studies pose a number of unique obstacles to achieve sufficient statistical power. In this paper, we developed an iterative, univariate probability-based approach to outlier detection that addresses many of the challenges. A challenge that may need more attention is how to accurately detect multiple outliers. Since *iLOO* identifies outliers by sequentially omitting feature-level observations, *iLOO* is likely to mask one outlier with another if the outliers are clustered together. However, one may argue that several neighboring extreme observations may not be artifacts but true expression and should not be classified as outliers, particularly in small-sample problems. By making use of information inherent in a given RNA-seq study (e.g. empirical distribution and sequencing depth), the proposed method minimizes the prevalence of false positive calls and maximizes true outlier detection. Advantages of the iterative approach over existing methodologies were demonstrated in real data and simulated data and were observed in sample sizes as small as 5.

## Supporting Information

S1 Fig Venn diagram of the number of single outlier features detected by *iLOO* and *edgeR-robust*. The totals provided present the number of single outlier features identified by *iLOO* and *edgeR-robust* in the Wang et al. dataset.
(DOC)

S2 Fig Scatterplot of read counts observed in real data for a sample of features. Scatterplot of raw counts for five representative features displaying counts identified as outliers by *iLOO* (purple diamond), *edgeR-robust* (red diamond), and both methods (blue diamond) in the Wang et al. dataset.
(DOC)

S1 R Code The R code for running iLOO. (DOC)

S1 Table Outliers detected in the Wang et al. dataset. Number of features with 0, 1, and 2 detected outliers.
(DOC)

S2 Table Mean (standard deviation) of accuracy metrics for simulated RNA-seq data comparing *iLOO* (using *DESeq2* normalized read counts) to DESeq2. Summary statistics for total accuracy, outlier accuracy, and non-outlier accuracy are provided for the simulation study comparing *iLOO* (using *DESeq2* normalized read counts) to *DESeq2*. A full simulation study was carried out for each sample size setting consisting of 5, 10, 15, 20, 30, and 40 replicates.
(DOC)
