# Supplementary material for: An Iterative Leave-One-Out Approach to Outlier Detection in RNA-Seq Data
Source: PLoS One. 2015 Jun 3;10(6):e0125224. doi: 10.1371/journal.pone.0125224 (PMC4454687; doi:10.1371/journal.pone.0125224)
Supplement: S1 Fig — The totals provided present the number of single outlier features identified by iLOO and edgeR-robust in the Wang et al. dataset. (DOC) [file pone.0125224.s001.doc]

**Supplementary Information**

“An iterative leave-one-out approach to outlier detection in RNA-seq data”
Nysia I. George, John F. Bowyer, Nathaniel M. Crabtree, and Ching-Wei Chang


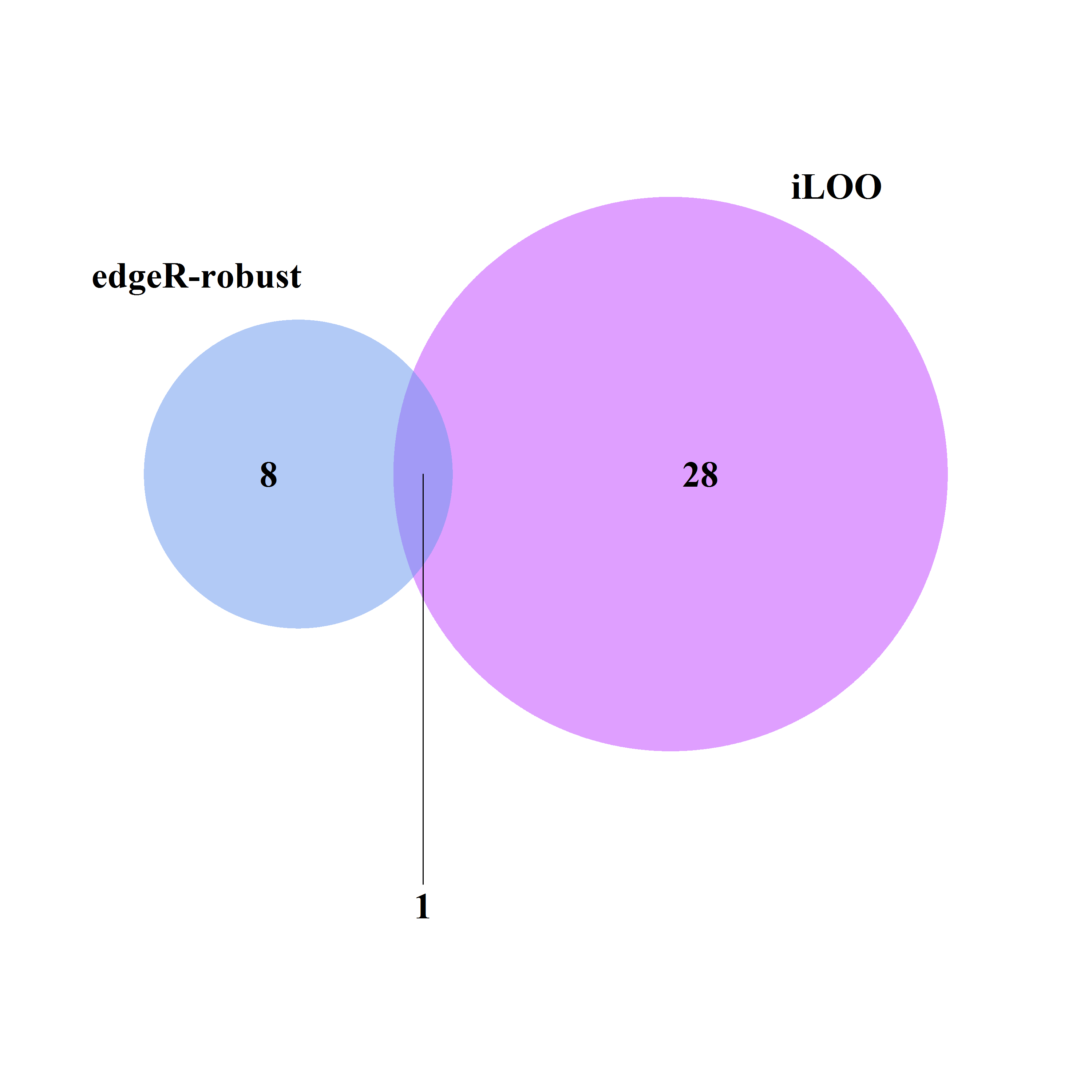


# S1 Fig.. Venn diagram of the number of single outlier features detected by *iLOO* and *edgeR-robust*. The totals provided present the number of single outlier features identified by *iLOO* and *edgeR-robust* in the Wang et al. dataset.
