# Supplementary material for: An Iterative Leave-One-Out Approach to Outlier Detection in RNA-Seq Data
Source: PLoS One. 2015 Jun 3;10(6):e0125224. doi: 10.1371/journal.pone.0125224 (PMC4454687; doi:10.1371/journal.pone.0125224)
Supplement: S2 Fig — Scatterplot of raw counts for five representative features displaying counts identified as outliers by iLOO (purple diamond), edgeR-robust (red diamond), and both methods (blue diamond) in the Wang et al. dataset. (DOC) [file pone.0125224.s002.doc]

**Supplementary Information**

“An iterative leave-one-out approach to outlier detection in RNA-seq data”
Nysia I. George, John F. Bowyer, Nathaniel M. Crabtree, and Ching-Wei Chang

#

**S2 Fig..** **Scatterplot of read counts observed in real data for a sample of features.** Scatterplot of raw counts for five representative features displaying counts identified as outliers by *iLOO* (purple diamond), *edgeR-robust* (red diamond), and both methods (blue diamond) in the Wang et al. dataset.
