# Supplementary material for: An Iterative Leave-One-Out Approach to Outlier Detection in RNA-Seq Data
Source: PLoS One. 2015 Jun 3;10(6):e0125224. doi: 10.1371/journal.pone.0125224 (PMC4454687; doi:10.1371/journal.pone.0125224)
Supplement: S1 R Code — (DOC) [file pone.0125224.s003.doc]

**Supplementary Information**

“An iterative leave-one-out approach to outlier detection in RNA-seq data”
Nysia I. George, John F. Bowyer, Nathaniel M. Crabtree, and Ching-Wei Chang

**R code for iLOO**

Note: The function *iLOO* requires an input $g\times n$matrix, which represents a matrix of read counts for a treatment or homogeneous group containing $n$ samples and $g$features. *iLOO* returns a $g\times n$matrix, where only the outlier read counts are present (all non-outlier values have been NA’ed).

iLOO <- function(sub) {

#estimate sequencing depth and compute cutoff

sd <- mean(apply(sub,2,sum,na.rm=T))
sdcut <- 1/sd

#implement iterative scheme

outl <- apply(sub, 1, function(z) {

x <- z

nbp <- 0

out <- rep(0,0)

track <- c(1:length(x))

#iterative scheme

while((min(nbp,na.rm=T) < sdcut) & (length(x)>2)) {

#build matrix with rows representing leave-one out observation

mat <- matrix(rep(x,length(x)),ncol=length(x),byrow=T)

diag(mat) <- NA

tmp <- t(mat)

mat <- t(matrix(tmp[!is.na(tmp)],nrow=(length(x)-1),ncol=(length(x))))

#fit negative binomial or Poisson distribution

nbfit <- apply(mat,1,function(y) {

if(length(y)>1) {

v <- var(y)

m <- mean(y)

if(all(y==0)=="TRUE") {

output <- NA

} else if (v>m) {

p <- mean(y)/var(y)

r <- mean(y)^2/(var(y)-mean(y))

output <- c(p,r)

} else {

lamb <- mean(y)

output <- c(lamb)

}

} else output <- NA

list(output)

})

nbfit <- lapply(nbfit, "[[", 1)

#compute probabilities for leave-one out observation

nbp <- rep(0,0)

for (i in 1:length(nbfit)) {

if(length(nbfit[[i]])==2) {

nbp <- c(nbp,dnbinom(x[i],prob=nbfit[[i]][1],size=nbfit[[i]][2]))

} else {

nbp <- c(nbp,dpois(x[i],lambda=nbfit[[i]][1]))

}

}

#compare probabilities to cutoff

sel <- which(nbp < sdcut)

if(length(sel)>0) x <- x[-sel]

if(length(out)==0) {

out <- c(out,track[sel])

} else {

out <- c(out,track[-out][sel])

}

fout <- rep(NA,length(z))

if(length(out)>0) {

fout[out] <- z[out]

}

}

list(fout)

})

#new data matrix with outliers (all other data is NA’ed)

identout <- matrix(unlist(outl),nrow=nrow(sub),ncol=ncol(sub),byrow=T)

colnames(identout) <- colnames(sub)

rownames(identout) <- rownames(sub)

return(identout)

} #end of iLOO function
