# Supplementary material for: An Iterative Leave-One-Out Approach to Outlier Detection in RNA-Seq Data
Source: PLoS One. 2015 Jun 3;10(6):e0125224. doi: 10.1371/journal.pone.0125224 (PMC4454687; doi:10.1371/journal.pone.0125224)
Supplement: S1 Table — Number of features with 0, 1, and 2 detected outliers. (DOC) [file pone.0125224.s004.doc]

**Supplementary Information**

“An iterative leave-one-out approach to outlier detection in RNA-seq data”
Nysia I. George, John F. Bowyer, Nathaniel M. Crabtree, and Ching-Wei Chang

# S1 Table. Outliers detected in the Wang et al. dataset.

| **No. of Outliers** | ***iLOO*** | ***edgeR-robust*** |
| --- | --- | --- |
| **0** | 11051 | 11072 |
| **1** | 29 | 9 |
| **2** | 1 | 0 |

Number of features with 0, 1, and 2 detected outliers.
