# Supplementary material for: An Iterative Leave-One-Out Approach to Outlier Detection in RNA-Seq Data
Source: PLoS One. 2015 Jun 3;10(6):e0125224. doi: 10.1371/journal.pone.0125224 (PMC4454687; doi:10.1371/journal.pone.0125224)
Supplement: S2 Table — Summary statistics for total accuracy, outlier accuracy, and non-outlier accuracy are provided for the simulation study comparing iLOO (using DESeq2 normalized read counts) to DESeq2. A full simulation study was carried out for each sample size setting consisting of 5, 10, 15, 20, 30, and 40 replicates. (DOC) [file pone.0125224.s005.doc]

**Supplementary Information**

“An iterative leave-one-out approach to outlier detection in RNA-seq data”
Nysia I. George, John F. Bowyer, Nathaniel M. Crabtree, and Ching-Wei Chang

**S2 Table.** Mean (standard deviation) of accuracy metrics for simulated RNA-seq data comparing *iLOO* (using *DESeq2* normalized read counts) to *DESeq2*.

| **Method** | **Accuracy** | **Sample Size** | | | | | |
| --- | --- | --- | --- | --- | --- | --- | --- |
|  |  | **5** | **10** | **15** | **20** | **30** | **40** |
| ***iLOO*** | *Total* | 0.9680 (0.0381) | 0.9812 (0.0215) | 0.9890 (0.0158) | 0.9930 (0.0069) | 0.9938 (0.0092) | 0.9945 (0.0066) |
|  | *Outlier* | 0.9250 (0.0737) | 0.9607 (0.0401) | 0.9765 (0.0314) | 0.9857 (0.0151) | 0.9882 (0.0190) | 0.9912 (0.0105) |
|  | *Non-Outlier* | 0.9728 (0.0343) | 0.9835 (0.0195) | 0.9904 (0.0141) | 0.9938 (0.0060) | 0.9944 (0.0082) | 0.9949 (0.0062) |
| ***DESeq2*** | *Total* | 0.9205 (0.0175) | 0.9275 (0.0713) | 0.9400 (0.0868) | 0.9704 (0.0353) | 0.9716 (0.0808) | 0.9849 (0.0510) |
|  | *Outlier* | 0.2897 (0.0516) | 0.5280 (0.0885) | 0.6504 (0.1035) | 0.7899 (0.0899) | 0.8805 (0.0822) | 0.9354 (0.0531) |
|  | *Non-Outlier* | 0.9906 (0.0231) | 0.9719 (0.0793) | 0.9722 (0.0938) | 0.9905 (0.0374) | 0.9818 (0.0840) | 0.9904 (0.0533) |

Summary statistics for total accuracy, outlier accuracy, and non-outlier accuracy are provided for the simulation study comparing *iLOO* (using *DESeq2* normalized read counts) to *DESeq2.* A full simulation study was carried out for each sample size setting consisting of 5, 10, 15, 20, 30, and 40 replicates.
